# Prognostic Impact of Low-Level p53 Expression on Brain Astrocytomas Immunopositive for Epidermal Growth Factor Receptor

**DOI:** 10.3390/cimb44090284

**Published:** 2022-09-09

**Authors:** Hung-Pei Tsai, Chien-Ju Lin, Chieh-Hsin Wu, Yi-Ting Chen, Ying-Yi Lu, Aij-Lie Kwan, Ann-Shung Lieu

**Affiliations:** 1 Division of Neurosurgery, Department of Surgery, Kaohsiung Medical University Hospital, Kaohsiung City 807, Taiwan; 2 School of Pharmacy, College of Pharmacy, Kaohsiung Medical University, Kaohsiung City 807, Taiwan; 3 Department of Surgery, School of Medicine, College of Medicine, Kaohsiung Medical University, Kaohsiung City 807, Taiwan; 4 Department of Pathology, Kaohsiung Medical University Hospital, Kaohsiung City 807, Taiwan; 5 Department of Pathology, School of Medicine, College of Medicine, Kaohsiung Medical University, Kaohsiung City 807, Taiwan; 6 Department of Dermatology, Kaohsiung Veterans General Hospital, Kaohsiung City 807, Taiwan; 7 Cosmetic Applications and Management Department, Yuh-Ing Junior College of Health Care & Management, Kaohsiung City 807, Taiwan; 8 Graduate Institute of Medicine, College of Medicine, Kaohsiung Medical University, Kaohsiung City 807, Taiwan; 9 Department of Neurosurgery, University of Virginia, Charlottesville, VA 22903, USA

**Keywords:** astrocytoma, epidermal growth factor receptor, p53, prognosis

## Abstract

Although the expression of p53 and epidermal growth factor receptor (EGFR) is associated with therapeutic resistance and patient outcomes in many malignancies, the relationship in astrocytomas is unclear. This study aims to correlate p53 and EGFR expression in brain astrocytomas with overall patient survival. Eighty-two patients with astrocytomas were enrolled in the study. Semi-quantitative p53 and EGFR immunohistochemical staining was measured in tumor specimens. The mean follow-up after astrocytoma surgery was 18.46 months. The overall survival rate was 83%. Survival was reduced in EGFR-positive patients compared with survival in EGFR-negative patients (*p* < 0.05). However, no significant differences in survival were detected between patients with high and low p53 expression. In patients with low p53 expression, positive EGFR staining was associated with significantly worse survival compared with patients with negative EGFR staining (log-rank test: *p* < 0.001). Survival rates in positive and negative EGFR groups with high p53 protein expression were similar (log-rank test: *p* = 0.919). The IC50 of an EGFR inhibitor was higher in GBM cells with high p53 protein expression compared with the IC50 in cells with low p53 expression. Combined EGFR and p53 expression may have prognostic significance in astrocytomas.

## 1. Introduction

Astrocytomas, which are the most common primary malignant tumors of the central nervous system in adults, consist of neoplastic, star-shaped glial cells. Pathologically, astrocytomas are classified as grade I to IV by the World Health Organization (WHO), according to the degree of differentiation, cellular mitosis, necrosis, atypia, and angiogenesis [1]. Low-grade and high-grade astrocytomas are classified by the WHO as grades I–II and III–IV, respectively, to describe dichotomous tumor behaviors. Although surgical resection of brain astrocytomas provides timely relief of the mass effect, high-grade astrocytomas unavoidably recur even after concurrent chemoradiotherapy. With advances in targeted therapy, oncolytic viral therapy, and immunotherapy, tumor resection with adjuvant chemotherapy and radiation is the standard therapy for gliomas [2,3]. The currently adopted histological grading for tumors is not predictive of the treatment response or patient outcome. Therefore, the identification of other predictors of prognosis is essential for guiding appropriate treatment strategies. Gliomas are identified more and more by molecular pathology according to The 2021 WHO Classification of Tumors of the Central Nervous System, including isocitrate dehydrogenase1/2 (IDH1/2), 1p/19q-codeleted, phosphatase and tensin homolog (PTEN) alterations, telomerase reverse transcriptase promoter gene mutations, EGFR amplification and mutation, and TP53 (which encodes the p53 tumor suppressor protein) mutation [3,4]. EGFR amplification and TP53 mutation are the most common genetic alterations in glioblastomas (GBMs).

Epidermal growth factor receptor (EGFR) is one of the most frequently discussed prognostic indicators. EGFR gene amplification and overexpression, which are particularly striking features of GBMs, are associated with worse outcomes in patients with GBMs [5,6]. GBMs, which are classified as WHO grade IV astrocytomas, comprise approximately 40% of tumors [7]. Although pharmacological treatments targeting EGFR or its downstream signaling pathway have been investigated, no significant benefit has been noted in unselected patients with high-grade astrocytomas [8,9,10]. The tumorigenesis of astrocytomas is extremely complex, involving various key biomarkers, including p53 [11,12]. The p53 protein, which is encoded by the TP53 tumor suppressor gene, exerts regulatory effects on the cell cycle and DNA repair, and it modulates the resistance to EGFR inhibitors in lung cancer cells [13]. However, the potential clinical significance of concomitant expressions of EGFR and p53 has not been addressed. Therefore, the present study aims to determine the prognostic significance of concomitant expressions of EGFR and p53 in patients with astrocytomas.

## 2. Materials and Methods

### 2.1. Study Population and Protocol

Data from the medical records of 144 patients who underwent surgery for brain astrocytomas at a tertiary referral medical center (Kaohsiung Medical University Hospital, Taiwan) were retrospectively collected, including patient demography, WHO tumor grading [1], tumor size, and overall survival. The inclusion criterion was histologically confirmed for astrocytomas within the study period. Patients were excluded from the study if they underwent biopsy only, their complete data were not available, or their pathological examinations were of poor quality. Finally, data from 82 patients were analyzed. The paraffin-embedded astrocytoma specimens from the patients were processed and stained for EGFR and p53 using immunohistochemical staining. The results were correlated with the demographic information and prognostic patient outcomes. The study protocol was reviewed and approved by the institutional review board of Kaohsiung Medical University (KMUHIRB-E(II)-20180194).

### 2.2. Immunohistochemical Staining of p53 and EGFR

Specimen tissue blocks, which were previously fixed with 10% buffered formalin and embedded in paraffin, were sliced into 3 μm thick sections. The sections were deparaffinized, rehydrated, and autoclaved at 121 °C for 10 min in Target Retrieval solution at pH 6.0 (S2369, Dako, Santa Clara, CA, USA). Twenty minutes later, endogenous peroxidase activity was blocked with 3% hydrogen peroxide for 5 min at room temperature. After washing with Tris buffer, the sections were incubated with p53 (1:600, M7001, Dako) or EGFR (1:200, NCL-L-EGFR-384, Leica, Wetzlar, Germany) antibodies for 1 h, followed by incubation with a secondary antibody (K5007, Dako) conjugated to horseradish peroxidase for 30 min. Finally, the slides were incubated in 3,3-diaminobenzidine for 5 min, counterstained with Mayer’s hematoxylin for 90 sec, and mounted with Malinol (2042, Muto, Tokyo, Japan). The expressions of p53 and EGFR were examined using a semi-quantitative scoring method. The sections were evaluated under a light microscope at ×100 magnification. The p53-immunostaining intensity was scored as 0 (negative), 1 (weak), 2 (moderate), and 3 (strong). A low expression and a high expression of p53 were defined by scores of 0–1 and 2–3, respectively. The immunostaining intensity of EGFR was scored as 0 (negative), 1 (weak), and 2 (strong), and a score of 1 or 2 represented positive EGFR expression.

### 2.3. Cell Culture and Treatment with the EGFR Inhibitor (AG490)

All the cells were incubated at 37 °C with 5% CO_2_. GBM8401, GB8901, and DBTRG-05MG cells were cultured in RPMI medium plus 10% fetal bovine serum (FBS). The U87-MG and SVGp12 cells were cultured in modified Eagle’s medium (MEM) plus 10% FBS. G5T cells were cultured in Dulbecco’s MEM medium plus 10% FBS. GBM8401, GBM8901, U87-MG, G5T, and DBTRG-05MG were GBM cell lines, and SVGp12 was a glial cell. The cells were treated with AG490 (S1143, Selleckchem, Houston, TX, USA) at 0, 100, 200, 300, 400, and 500 nM concentrations.

### 2.4. Proliferation Assay

GBM and glial cells were seeded at 1 × 10^4^ cells per 0.5 mL of medium per well in 24-well plates and cultured. At 24 h, the cells were incubated with 3-(4,5-dimethylthiazol-2-yl)-2,5-diphenyltetrazolium bromide (MTT) (M5655, Sigma–Aldrich, St. Louis, MO, USA) to detect cell proliferation, after treatment with AG490.

### 2.5. Western Blotting

Cells (3 × 10^5^ per well) were seeded in 6-well plates and lysed with 200 μL of RIPA buffer. Cell lysates (50 μg protein) were separated using 10% sodium dodecyl sulfate-polyacrylamide gel electrophoresis at 70 V for 30 min and 110 V for 1.5 h and, subsequently, transferred to PVDF membranes. After incubation for 1 h in blocking buffer, the membranes were incubated with primary antibodies (p531:500, M7001, Dako; EGFR, 1:200, NCL-L-EGFR-384, Leica; β-actin, 1:20,000, 60008-1-lg, Proteintech, Rosemont, IL, USA) for 16 h at 4 °C, followed by incubation with secondary antibodies (goat antirabbit, 1:5000, AP132P, Millipore, Billerica, MA, USA or goat antimouse, 1:5000, AP124P, Millipore) for 90 min. Enhanced chemiluminescence solution (Western Lightning, 205-14621, Perkin Elmer, Waltham, MA, USA) was used to detect specific protein bands, and the protein bands were analyzed using MiniChemi^TM^ imaging (Beijing Sage Creation, Beijing, China).

### 2.6. Statistical Analysis

The data were analyzed using SPSS 19.0 (Chicago, IL, USA). Categorical variables were compared using chi-square tests or Fisher’s exact tests. Kaplan–Meier survival curves were compared using log-rank tests. A one-way analysis of the variance was used to compare the Western blotting results. A *p*-value of <0.05 was considered statistically significant.

## 3. Results

### 3.1. General Characteristics

The 82 patients included in the analysis consisted of 45 (54.9%) males and 37 (45.1%) females. A total of 61 (74.4%) and 21 (25.6%) patients were <60 and >60 years old, respectively. Pathological examinations identified 31 (37.8%) patients with WHO grade I/II astrocytomas and 51 (62.2%) with WHO grade III/IV astrocytomas. A total of 36 (43.9%) patients had tumors that were >3 cm. In total, 37 (45.1%) patients had radiotherapy, and 28 (34.1%) patients had chemotherapy, such as temozolomide or bevacizumab.

### 3.2. Expressions of p53 and EGFR in Astrocytomas

**Figure 1:**
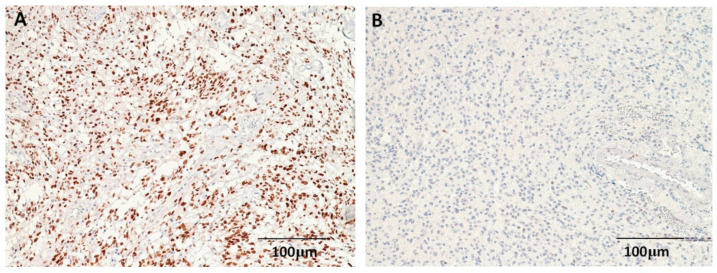
Immunohistochemical staining for p53 in astrocytomas. (**A**) High-level p53 expression in a glioblastoma patient. (**B**) Low-level p53 expression in a patient with grade II glioma (100×).

**Figure 2:**
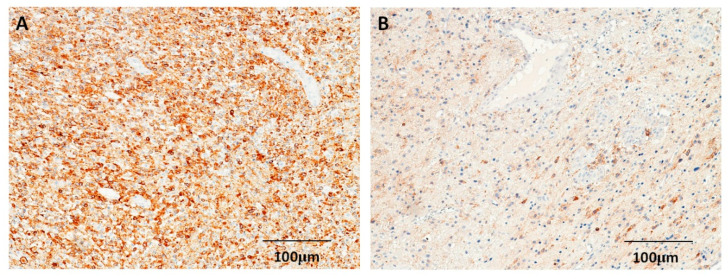
Immunohistochemical staining for EGFR in astrocytomas. (**A**) Astrocytoma with strong EGFR expression in a patient with glioblastoma. (**B**) Astrocytoma with weak EGFR expression in a patient with grade III tumor (100×).

Positive p53 staining was observed mostly in the nucleus (Figure 1). A total of 31 (37.8%) astrocytomas exhibited low p53 protein expression, and 51 (62.2%) astrocytomas exhibited high p53 protein expression. The only difference in the demographic and pathological features of the low-level (scores 0–1) and high-level (scores 2–3) p53-expression groups was in the WHO tumor grading (*p* = 0.022) (Table 1). Positive expressions of EGFR were observed mostly in the tumor cell membranes (Figure 2); in total, 54 (65.9%) of the 82 astrocytomas were positive for EGFR. No significant differences in any demographic or pathological features were detected between EGRF-positive and EGRF-negative astrocytomas (Table 1).

### 3.3. Survival Analysis According to p53 and EGFR Expression

**Figure 3:**
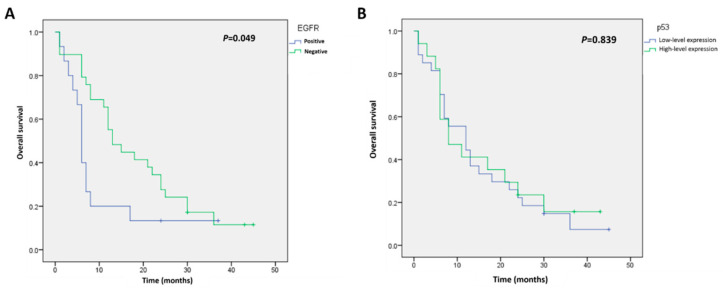
Kaplan–Meier analysis of overall survival in patients with astrocytomas according to (**A**) EGFR expression or (**B**) p53 expression.

**Figure 4:**
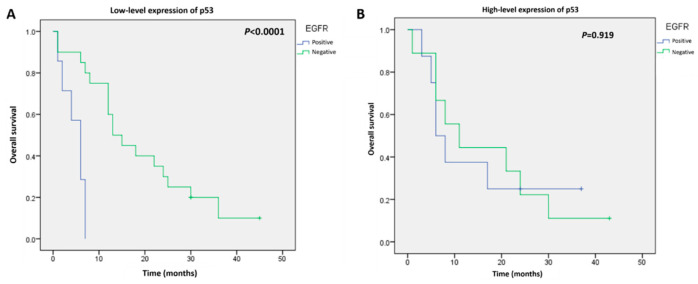
Kaplan–Meier analysis of overall survival in patients with EGFR-positive or EGFR-negative astrocytomas and (**A**) low-level expression of p53 or (**B**) high-level expression of p53.

The follow-up after astrocytoma surgery ranged from 1 to 60 months, and the overall survival rate was 83% (69/82 patients) at the last follow-up. The Kaplan–Meier curves for survival were significantly different in patients with positive versus negative EGFR immunostaining (log-rank test: *p* = 0.049) (Figure 3A). However, no significant differences between the patients with low-level and high-level p53 expression were detected (log-rank test: *p* = 0.839) (Figure 3B). To determine the value of combining these two biomarkers for predicting survival, subgroup analyses were performed. In the low-level p53 expression group, survival was remarkably worse in patients with positive EGFR astrocytomas compared with survival in patients with negative EGFR expression (log-rank test: *p* < 0.001) (Figure 4A). The Kaplan–Meier curves for survival were similar for EGFR-positive and -negative groups in patients with high p53 protein expression (log-rank test: *p* = 0.919) (Figure 4B).

### 3.4. Expression of p53, EGFR, and IC50s after Treatment with EGFR Inhibitors in GBM Cells

**Figure 5:**
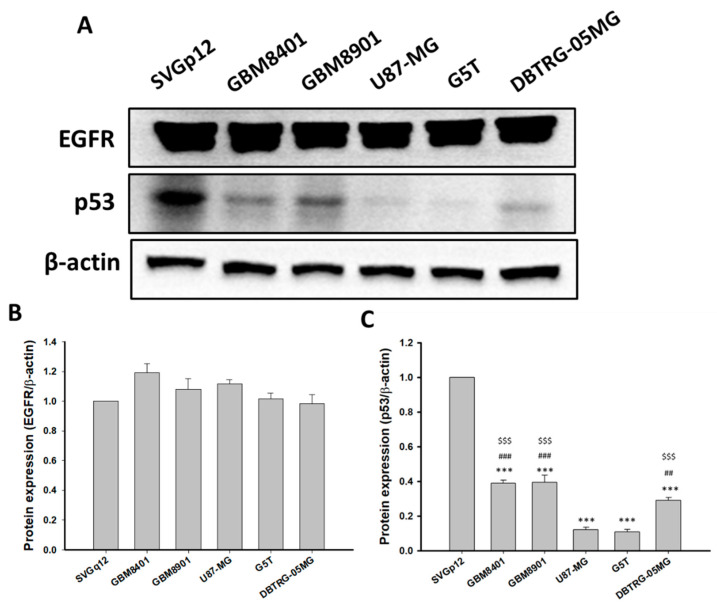
EGFR and p53 protein expressions in all tested astrocytoma cell lines (n = 6). (**A**) Western blot of EGFR and p53. (**B**) Relative EGFR protein expression levels. (**C**) Relative p53 protein expression levels. Expression levels were normalized to p53 expression in SVGp12 cells. *** *p* < 0.001 compared with the SVGp12. ^##^ *p* < 0.01 and ^###^ *p* < 0.001 compared with the U87-MG. ^$$$^ *p* < 0.001 compared with G5T.

Differences in p53 and EGFR protein expression in normal and cancer cells were determined by Western blotting in glial cells (SVGp12) and GBM cells (GBM8041, GBM8901, U87-MG, G5T, and DBTRG-05MG). No significant differences in EGFR protein expression were detected in GBM8401, GBM8901, U87-MG, G5T, DBTRG-05MG, or SVGp12 cells (Figure 5). However, p53 protein levels were significantly lower in GBM8401, GBM8901, U87-MG, G5T, and DBTRG-05MG cells compared to the expression in SVGp12 cells (Figure 5). In addition, p53 protein expression levels in GBM8401, GBM8901, and DBTRG-05MG were significantly higher than expression levels in U87-MG and G5T cells (Figure 5). Therefore, GBM8401, GBM8901, and DBTRG-05MG cells had high p53 expression levels, and U87-MG and G5T cells had low levels of p53 expression.

**Figure 6:**
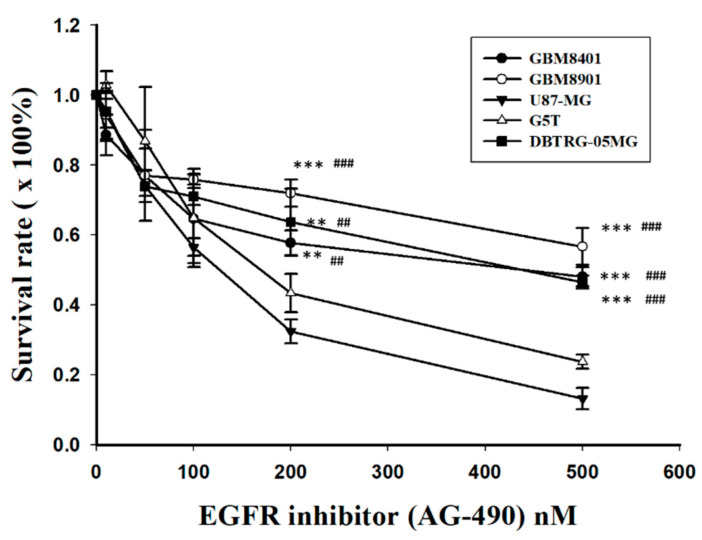
Cell viability analysis after AG490 treatment in all GBM cell lines (n = 4). ** *p* < 0.01 and *** *p* < 0.001 compared with U87-MG. ^##^ *p* < 0.01 and ^###^ *p* < 0.001 compared with G5T.

To compare the sensitivity or resistance to EGFR inhibition, all GBM cell lines were treated with AG490, an EGFR inhibitor, and the IC50s (drug concentration at 50% inhibition) were determined using a MTT assay. Following different doses, the cell viability of GBM8401, GBM8901, and DBTRG-05MG were significantly higher than that of U87-MG and G5T after treatment with AG490 at 200 nM and 500 nM (Figure 6). The IC50 values were 487, 620.25, 320.83, 363.41, and 510.7 nM for GBM8401, GBM8901, U87-MG, G5T, and DBTGR-05MG, respectively. The IC50 values for the EGFR inhibitor were higher in GBM cells with higher p53 protein expression (GBM8401, GBM8901, and DBTRG-05MG) compared with the IC50s in GBM cells with lower p53 protein expression (U87-MG and G5T).

## 4. Discussion

Astrocytomas have a broad spectrum of biological features, resulting in highly variable clinical prognoses. For patients with low-grade tumors, the median survival time is approximately 8 years [14]. However, the life expectancy of individuals with glioblastomas, an extremely malignant form of astrocytoma, is limited to 14 months, even with temozolomide treatment [15]. Many prognosticators for tumors are available, including age, pre-existing neurological deficits, histopathological features, tumor size, the degree of the surgical resection, and adjuvant chemoradiotherapy [14,16,17,18]. In addition, an increasing number of biomarkers for astrocytomas aid in predicting outcomes and guiding therapy. Despite the success in correlating O-6-methylguanine-DNA-methyltransferase (MGMT) methylation with survival in glioblastoma patients treated with alkylating agents [19], the clinical utility of other biomarkers is limited. In this study, we demonstrate that the concomitant status of EGFR and p53 is a key determinant of patient outcomes; survival was shorter in patients with EGFR-immunopositive astrocytomas with low p53 expression levels.

Most molecular markers have been investigated separately and show distinct roles in the pathogenesis of astrocytomas [11,12,20]. Nevertheless, our understanding of tumorigenesis is incomplete, and non-significant study results for individual molecular markers cannot be interpreted as unrelated to the disease. In a series of lung cancer patients, Yamaguchi et al. reported that EGFR, in concert with p53 mutations, affected tumor development and induced therapeutic resistance [21]. Moreover, the response of lung cancer cells to EGFR inhibitors and radiation could be attenuated by changes in p53 expression, as shown in p53 knockdown in parental cells and p53 functional restoration in resistant cells [13]. The EGFR status may predict radioresistance in non-small lung cancers, and normofractionated radiotherapy may need to be adjusted after an early response assessment [22]. In astrocytoma patients, EGFR and p53 expressions may also be related, and the biomarker associations should be recognized.

Our results demonstrated that the outcomes of astrocytoma patients were not significantly different in patients with low or high levels of p53 expression. The effects of p53 status alone on astrocytomas are controversial. In a retrospective study of patients after chemoradiotherapy for glioblastoma, p53 overexpression was positively associated with progression-free survival [23]. In contrast, an analysis of 220 glioblastoma patients did not find a correlation between p53 expression and median survival [24]. Another study was similarly unable to demonstrate the predictive value of TP53 mutations for the overall survival of primary glioblastoma patients [25]. According to the Cancer Genome Atlas, >70% of glioblastomas harbor somatic alterations in the p53 pathway [26]. However, the types and manifestations of p53 mutations are heterogeneous, and alterations may cause the loss of function, gain of function, or dominant-negative mutational effects in p53 [27]. Additionally, interactions with other mediators in the p53 signaling route are complicated and may partially explain why the role of p53 in the pathogenesis of astrocytomas remains inconclusive.

EGFR, a tyrosine kinase, consists of a transmembrane glycoprotein with an extracellular ligand-binding and a cytoplasmic domain. EGFR signaling regulates cell metabolism, multiplication, and survival. Approximately 50–60% of primary glioblastomas exhibit genetic changes in EGFR, including amplifications, rearrangements, alternative splicing, and mutations [28]. Similar to p53, some studies demonstrate that EGFR is a significant prognosticator for glioblastoma patients, while other studies demonstrate that EGFR is not relevant to predicting outcomes [5,11]. Interestingly, Ruano et al. reported that EGFR expression was associated with outcomes in a subgroup of glioblastoma patients according to p53 status [29]. Their data, which is inconsistent with our results, show that simultaneous immunopositivity of EGFR and p53 predicts worse survival. This discrepancy may be due to different patient cohorts; our study included all types of astrocytomas instead of only glioblastoma, and the age distribution and the ratio of immunopositive p53 patients were different. Theoretically, growth messages from overexpressed EGFR precipitate uncontrolled tumor proliferation, and alterations in p53 result in the loss of cell cycle brakes. Ultimately, increased growth stimuli combined with decreased cell death would strengthen the aggressiveness of astrocytomas and worsen the prognosis.

EGFR is a valuable potential target for treating malignant astrocytomas because EGFR exerts activity at the top of a signaling cascade to mediate critical cellular functions. Unfortunately, the clinical results for EGFR-targeted treatment have been disappointing. In a phase 1/2 trial, the median survival of newly diagnosed glioblastoma patients who underwent radiation therapy with gefitinib, an EGFR inhibitor, was similar to the survival of the control group receiving radiation alone [8]. Two other clinical studies showed that treatment of recurrent malignant gliomas with erlotinib, a receptor tyrosine kinase inhibitor of EGFR, had no benefits compared with control regimens [9,10]. Regarding the ineffective EGFR-aimed therapy, Azuaje et al. considered that EGFR-driven signaling should be regarded as an integrative interaction network rather than as a traditional linear pathway [30]. Therefore, our findings may be used to select candidates suitable for targeted treatment. In future studies, assessing the p53 status in EGFR-positive astrocytomas before initiating anti-EGFR therapy may improve the treatment response.

## 5. Conclusions

The simultaneous status of p53 and EGFR plays a key role in the prognosis of patients with brain astrocytomas. The stratification of these patients according to the inherent differences in these biomarkers is necessary to develop therapeutic protocols targeting EGFR in tumors.

## Figures and Tables

**Table 1:** Correlation of p53/EGFR expression with clinicopathologic parameters in astrocytomas.

	No.	p53 Expression (n, %)			EGFR Expression (n, %)		
		Low	High	*p*-Value	Negative	Positive	*p*-Value
**Age**				0.207			0.425
≤60	61	40 (48.8%)	21 (25.6%)		20 (24.4%)	41 (50%)	
>60	21	11 (13.4%)	10 (12.2%)		8 (9.8%)	13 (15.9%)	
**Gender**				0.412			0.09
Male	45	27 (32.9%)	18 (22.0%)		12 (14.6%)	33 (40.2%)	
Female	37	24 (29.3%)	13 (15.9%)		16 (19.5%)	21 (25.6%)	
**WHO Grade**				0.022 *			0.329
I/II	31	24 (29.3%)	7 (8.5%)		12 (14.6%)	19 (23.2%)	
III/IV	51	27 (32.9%)	24 (29.3%)		16 (19.5%)	35 (42.7%)	
**Tumor size**				0.519			0.150
≤3 cm	46	29 (35.4%)	17 (20.7%)		13 (15.9%)	33 (40.2%)	
>3 cm	36	22 (26.8%)	14 (17.1%)		15 (18.3%)	21 (25.6%)	
**Radiotherapy**				0.412			0.299
Yes	37	24 (29.3%)	13 (15.9%)		11 (13.4%)	26 (31.7%)	
No	45	27 (32.9%)	18 (22.0)		17 (20.7%)	28 (34.1%)	
**Chemotherapy**				0.329			0.065
Yes	28	16 (19.5%)	12 (14.6)		6 (7.3%)	22 (31.7%)	
No	54	35 (42.7%)	19 (23.2%)		17 (20.7%)	28 (34.1%)	
**KPS**				0.412			0.120
≤70	58	37 (45.1%)	21 (25.6%)		17 (20.7%)	41 (50%)	
>70	24	14 (17.1%)	10 (12.2%)		11 (13.4%)	13 (15.9%)	

* Statistically significant (*p* < 0.05).

## Data Availability

Not applicable.
